# A Novel Thermal Sensor for the Sensitive Measurement of Chemical Oxygen Demand

**DOI:** 10.3390/s150820501

**Published:** 2015-08-19

**Authors:** Na Yao, Zhuan Liu, Ying Chen, Yikai Zhou, Bin Xie

**Affiliations:** 1 Institute of Environmental Medicine, Tongji Medical College, Huazhong University of Science and Technology, Wuhan 430030, China; E-Mails: 59040368yaona@sina.cn (N.Y.); liuzhuan813@163.com (Z.L.); 2 Jingzhou Central Hospital, Jingzhou 434026, China; 3 Yancheng Blood Center, Yancheng 224005, China; 4 Hubei Center for Disease Control and Prevention, Wuhan 430079, China; E-Mail: cy_cdc@sina.com; 5 Pure and Applied Biochemistry, Lund University, Box 124, Lund SE-22100, Sweden

**Keywords:** chemical oxygen demand, thermal sensor, flow injection analysis system, sodium hypochlorite

## Abstract

A novel rapid methodology for determining the chemical oxygen demand (COD) based on a thermal sensor with a flow injection analysis system was proposed and experimentally validated. The ability of this sensor to detect and monitor COD was based on the degree of enthalpy increase when sodium hypochlorite reacted with the organic content in water samples. The measurement results were correlated with COD and were compared against the conventional method using potassium dichromate. The assay required only 5–7 min rather than the 2 h required for evaluation by potassium dichromate. The linear range was 5–1000 mg/L COD, and the limit of detection was very low, 0.74 mg/L COD. Moreover, this method exhibited high tolerance to chloride ions; 0.015 mol/L chloride ions had no influence on the response. Finally, the sensor was used to detect the COD of different water samples; the results were verified by the standard dichromate method.

## 1. Introduction

Chemical oxygen demand (COD) is widely used as one of the most important parameters for the determination of water quality. It is defined as the number of oxygen equivalents consumed in the oxidation of organic compounds by powerful oxidizing agents such as dichromate or permanganate, thus indicating the organic content in the water sample [1]. Of the standard dichromate- or permanganate-based COD methods [2], the former is more commonly used mainly due to its higher oxidizing power and superior reproducibility [3,4]. However, the conventional titration method for evaluating COD has some intrinsic drawbacks. Above all, the analysis time is protracted (~2 h) because a time-consuming sample refluxing process is required to achieve nearly complete oxidation [5]. Additionally, the manipulations are complicated and the reproducibility of the results is largely dependent upon the skill of the operator [6]. Finally, expensive and highly toxic reagents (Ag_2_SO_4_ and HgSO_4_) are applied, which further contribute to severe environmental pollution and threaten human health [7,8]. The shortage of sustainable water resources and increasing awareness of environmental protection calls for an onsite, environmentally friendly, and rapid analytical method.

In recent years, numerous efforts have been exerted to solve the problems of the current methodologies and develop a simple, environmentally friendly, and rapid analytical method for COD detection. The new approaches have been based on either electrocatalytic [9,10,11,12] or photocatalytic [13,14,15,16] oxidation principles. Although they have demonstrated many advantages over the traditional COD methods, such as the rapidity of the analysis, the direct analytical acquisition, and the facile incorporation into on-line analysis monitoring systems, the reliability of these new methods is uncertain and they are not practical for use. This is mainly due to the fact that they are incapable of oxidizing a wide spectrum of organic compounds [17].

Our group has pioneered the development of the thermal sensor for the analysis of COD. In this approach, the sensor measures the heat released during the oxidation of organic compounds in a water sample [18,19,20]. Because the flow injection analysis (FIA) technique is used in the thermal sensor assay [21], control of the carrier solution and water sample is straightforward in this continuous analysis [22]. The peak height of the thermometric recording is proportional to the enthalpy change resulting from the oxidation of the organic content in the water [19,23]. In this study, we chose sodium hypochlorite (NaClO) as the oxidant after investigating its activity in addition to those of ozone, hydrogen peroxide, and Fenton’s reagent. These reagents, as opposed to sodium hypochlorite, generated excessive amounts of bubbles during glycine oxidation, leading to an increase in signal noise and an imbalance of the baseline. The carrier solution was re-distilled water. The flow rate of the mobile phase, the concentration, acidity, and stability of NaClO, and the signal amplification affect the sensor method for the determination of COD. A water sample containing glycine was chosen for optimization of the analytical parameters. In this study, re-distilled water was used as the blank water sample and experimental data were presented after correction against the blank value. The FIA-sensor was successfully used for both standard solutions and real water samples; the results were well correlated with COD values determined by the conventional potassium dichromate method. Thus, promise for the application of this method was demonstrated.

## 2. Experimental

### 2.1. Materials and Reagents

All reagents were of analytical grade and were used without further purification. NaClO (available chlorine >10%), H_2_SO_4_, NaOH, glycine, K_2_Cr_2_O_7_, Ag_2_SO_4_, HgSO_4_, and (NH_4_)_2_Fe(SO_4_)_2_ were purchased from the Sinopharm Group Chemical Reagent Co. Ltd. (Beijing, China). A stock solution of glycine (7.5 g/L) was prepared using redistilled water.

### 2.2. ET Instrument

**Figure 1:**
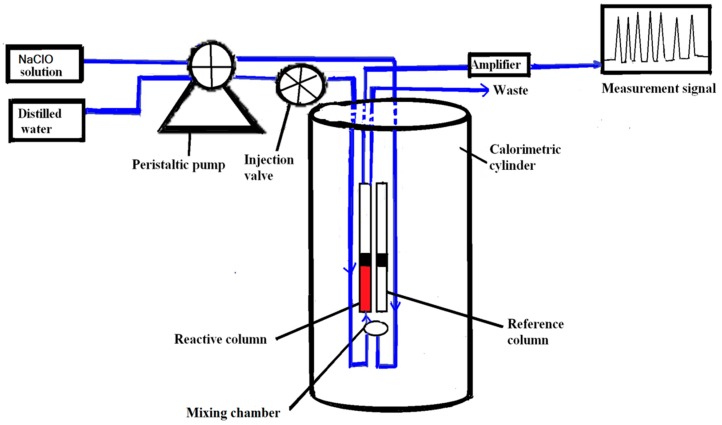
Schematic of the thermal sensor instrument for COD analysis.

A schematic of the FIA-thermal sensor for COD analysis is presented in Figure 1. The thermal sensor device consists of a peristaltic pump mounted with one tube for each channel, an injection valve, and an instrument (Omik Bioscience AB, Lund, Sweden). The instrument applied in this study was the same as the device used in the determination of antibiotics in whole blood by Bin Xie *et al.* [24] in 2013; it included two inlet flow channels, a reactant mixing chamber, reaction column, and one outlet channel. One inlet channel was used for the analyte and the other inlet was used for the NaClO solution. The reaction column was coupled with a thermistor for measurement and comparison with a reference column and thermistor for differential temperature measurements (see Figure 1). 

The flows of the redistilled water and NaClO solution were maintained using a peristaltic pump, generally operated at 1.0 mL/min. The water sample and NaClO solutions were introduced into a mixing chamber and the reactive column via a thermal heat exchanger installed inside the calorimeter. Then, the thermal signals were amplified through an amplifier (set at 100 scale), which was connected to a computer for data processing. The thermal control unit of the instrument was maintained at 30 °C through all the experiments. In this study, the signals were recorded in the form of peak heights (voltages). Sample volumes of 350 μL were injected using a six-port injection valve after thermal equilibration of the column. Fluids used in the investigations were degassed to avoid the trapping of air bubbles within the reaction column and thus reduce noise.

### 2.3. Conventional Dichromate Method

The conventional dichromate method was performed to determine COD values based on the Chinese National Standard GB 11914-89. A sample solution (20 mL) was added to a flask; and refluxed for 2 h in a thermostat-controlled bath at 165 °C. Then, the excess dichromate was determined by titration using (NH_4_)_2_Fe(SO_4_)_2_ as the titrant. Finally, the value of COD was calculated. For the 150 mg/L glycine solution, the COD value was measured to be 98.4 mg/L O_2_ using the conventional dichromate method, which was consistent with the theoretical value of 96 mg/L O_2_.

## 3. Results and Discussion

### 3.1. Assay Optimization

As defined, the COD value reflects the total content of the organic species in a water sample. For simplification, compounds such as glucose, glycine, or potassium hydrogen phthalate were used as the standard reagents. Here, our measurements were optimized for glycine, and each sample solution underwent three determinations. 

### 3.2. Effect of pH

**Figure 2:**
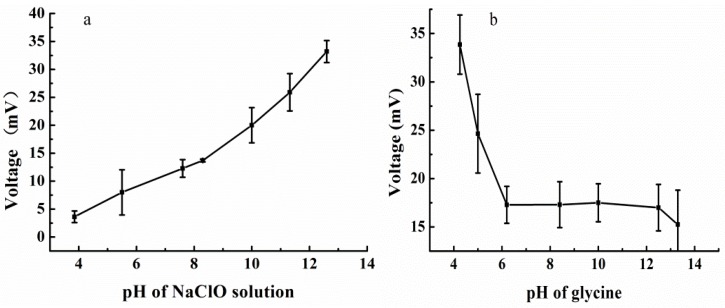
(**a**) Effect of pH of NaClO solution on the thermal sensor for a 350 μL injection of 156.2 mg/L glycine solution, at a 1.0 mL/min flow rate and signal magnification of 100; (**b**) pH effect of the glycine solution in the thermal sensor when injecting 350 μL of 156.2 mg/L glycine solution, at a 1.0 mL/min flow rate and signal magnification of 100.

The influence of the pH of the NaClO solution was studied. Measurements were carried out in the flow system with 156.2 mg/L glycine solution of pH 4.25 while varying the pH values of the NaClO solution. The results are presented in Figure 2a.

From the graph, it is evident that the response signal increased up with the increasing pH and reached the maximum value at 12.6. Significant drift from the baseline was noticed so that the determinations of COD couldn’t be obtained when the pH was greater than 12.6. On this basis, further experiments were carried out at pH 12.6. The effect of the pH of the glycine solution on the reaction was also examined, as shown in Figure 2b. The graph clearly suggests that a maximum signal was achieved at pH 4.25. The oxidation of glycine decreases at pH values between 4.25 and 13.3. This phenomenon may be due to the greater oxidation ability of the NaClO solution if the glycine solution is acidic. The next experiments were carried out with NaClO of pH 12.6 and glycine of pH 4.25.

### 3.3. Influence of Concentration of NaClO Solution

**Figure 3:**
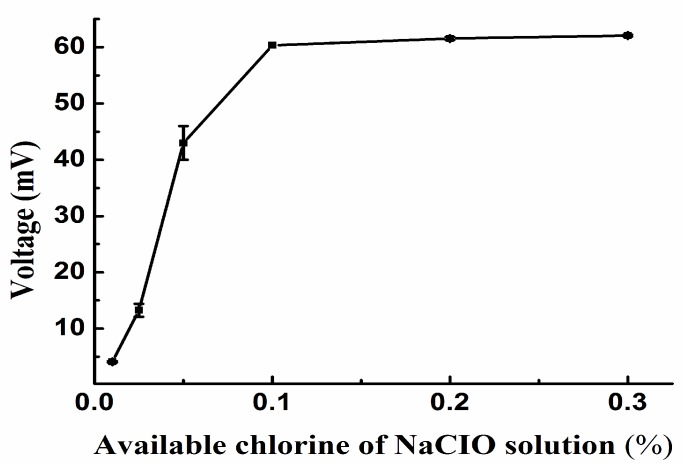
Peak voltage from the reaction of glycine with different NaClO concentrations (Other conditions are as in Figure 2).

NaClO solutions with different concentrations of available chlorine were introduced into the thermal sensor, and the resulting peak heights were examined. As displayed in Figure 3, the peak height for a 156.2 mg/L glycine solution significantly increased with available chlorine from 0% to 0.1%, and then changed only slightly from 0.1% to 0.3%. When the concentration of the NaClO solution was too high, the degradation intensified. In addition, highly concentrated NaClO can damage the instrument due to its strong corrosivity. Therefore, a NaClO solution containing 0.1% available chlorine was chosen as the experimental concentration.

### 3.4. Effect of Flow Rate

The effect of the flow rate on the thermometric response of the thermal sensor was also studied by applying various flow rates during the experiments. The thermal signals for a 156.2 mg/L glycine solution at different flow rates are listed in Table 1. When the flow rate was 0.25 mL/min, the baseline drift was sharp, as the evolved heat was distributed. At 1.5 mL/min, the results of three repeated measurements were notably different. When the flow rate was between 0.5 and 1.0 mL/min, the peaks were sharp and differences among the three determinations were small. Although the response of the thermal sensor is flow-dependent, it could be optimized to achieve a balance between sensitivity and response time. In this investigation, therefore, an optimal flow rate of 1.0 mL/ min was utilized.

**Table 1:** Influence of flow rate on the thermal sensor. (Other conditions were as in Figure 2)

Flow Rate (mL/min)	ΔV (mV)		RSD (%)	
0.25	0	0	0	0
0.5	27.4	25.8	24.6	5.417
1.0	35.1	36.1	35.3	1.491
1.5	25.6	29.4	19.2	20.842
2.0	22.0	25.1	30.3	16.256

### 3.5. Detection Limit and Linear Range

**Figure 4:**
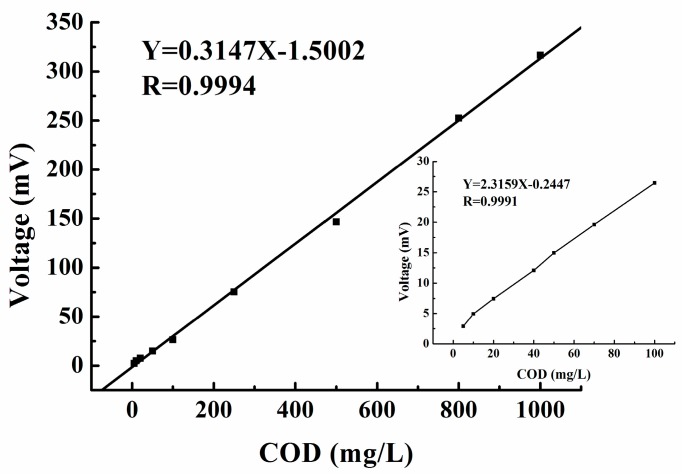
Calibration curve for standard glycine samples. Data represent the average of three measurements.

**Figure 5:**
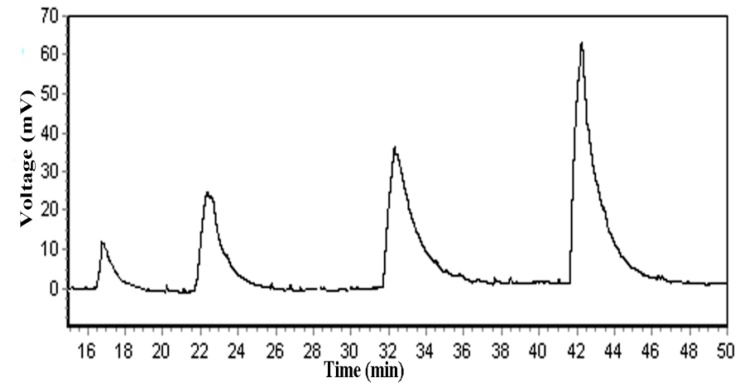
An actual sensor signal record for measurements of 50, 80, 100, and 200 mg/L COD solutions.

The detection limit and linear range were evaluated using sample solutions containing different concentrations of glycine. A real detection limit of 5 mg/L COD with a linear range up to 1000 mg/L COD (C, mg/L of O_2_) and regression coefficient of 0.999 could be achieved under the experimental conditions employed. The results are presented in Figure 4. An actual sensor signal record for measurements of 50, 80, 100, and 200 mg/L COD solutions is presented in Figure 5. The detection limit of the sensor was 0.74 mg/L for a signal-to-noise ratio of 3.

### 3.6. Reproducibility and Operational Stability

**Figure 6:**
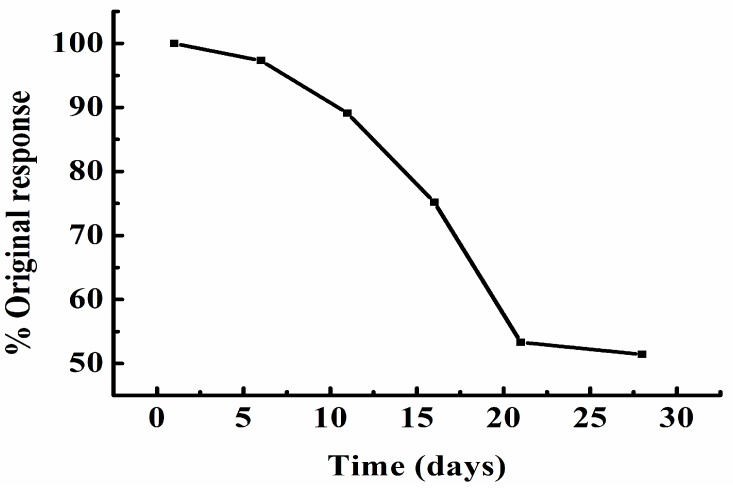
Stability of the response to 100 mg/L COD using FIA- thermal sensor observed for 28 continuous days. Samples were injected six times every day during this period.

The reproducibility of the instrument over successive detections was assessed by measuring the voltage signal at 50 mg/L COD. The relative standard deviation (RSD) was 3.4% for eleven determinations, suggesting excellent reproducibility. The stability of the instrument was tested by continuously monitoring the voltage response at a fixed COD concentration of 100 mg/L over a period of 28 d with 6 daily measurements. NaClO was stored in sealed, cool and dry condition. The results over the studied period are presented in Figure 6. After 4 d of operation, about 2.7% decay in the response was observed. The signal responses monitored at 10, 20, and 28 d were 89.1%, 53.3% and 51.4% of the original response, respectively, mainly because of the instability of the NaClO solution. NaClO was so volatile that it could lead to the reduction of its concentration. In conclusion, it is the best that daily prepared NaClO solutios must be used.

### 3.7. Interference

The interference of chloride ions must be considered when determining COD. Therefore, the influence of chloride ions on the thermal sensor detection of COD was investigated. In the presence of a 0.015 mol/L chloride solution, the voltage signal at 10 mg/L COD remained roughly unchanged, revealing that this sensor has a high tolerance for chloride ions.

### 3.8. Analytical Application

**Figure 7:**
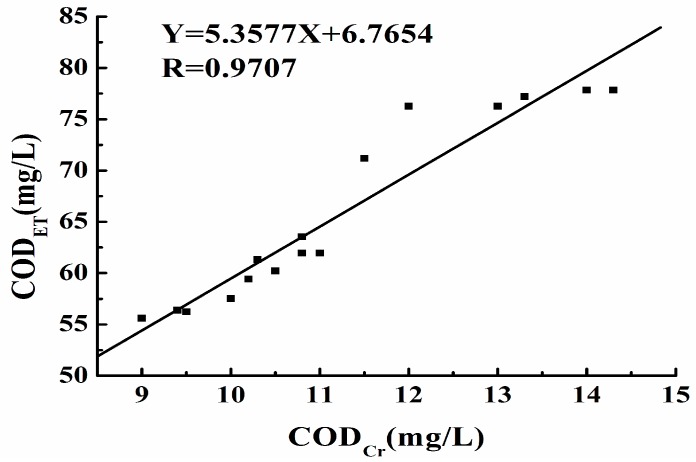
Correlation of COD values of real samples from rivers and lakes determined by the thermal sensor and conventional method. The COD_ET_ values of real samples were inserted into the calibration equation using the standard sample method, COD_ET_ = 6.7654 + 5.3577 COD_Cr_, R = 0.9707. Data represent the average of three measurements, n = 16.

To certify the performance of the thermal sensor in real sample analysis, it was used to detect COD values of actual water samples which were collected from different lakes and rivers including the Yangtze and Han Rivers in Wuhan City, China. The COD values of these water samples were also measured by the conventional dichromate method. Each sample solution underwent three determinations. Figure 7 illustrates the relationship between the results obtained by the proposed method and the conventional method for 16 water samples. 

## 4. Conclusions

It was found that the COD value detected by the dichromate titration method (denoted as COD_Cr_) was well correlated with that by the thermal sensor method (denoted as COD_ET_). The linear regression equation was COD_ET_ = 6.7654 + 5.3577 COD_Cr_, with a correlation coefficient of 0.9707, indicating the good agreement of the two methods. Therefore, this novel method is feasible and accurate for the detection of COD. The experimental results obtained in this study verify the proposed COD analytical method based on a flow-injected thermal sensor. The developed method was successfully applied in the determination of the CODs of standard and real samples. The method is environmentally friendly, robust, rapid, and easy to operate. More importantly, it requires only 5–7 min to complete an assay and consumes minimal reagents and sample. The thermal sensor method is especially useful for monitoring the COD variations in actual contaminated water samples, as it provides good quantification with high precision, as well as qualitative information, based on thermal stability.
